# Maternal Executive Functioning, Emotional Availability and Psychological Distress During Toddlerhood: A FinnBrain Birth Cohort Study

**DOI:** 10.3389/fpsyg.2021.735734

**Published:** 2021-10-08

**Authors:** Elisabeth Nordenswan, Kirby Deater-Deckard, Mira Karrasch, Matti Laine, Eeva-Leena Kataja, Eeva Holmberg, Eeva Eskola, Hetti Hakanen, Hasse Karlsson, Linnea Karlsson, Riikka Korja

**Affiliations:** ^1^The FinnBrain Birth Cohort Study, Turku Brain and Mind Center, Department of Clinical Medicine, University of Turku, Turku, Finland; ^2^Department of Psychology, Åbo Akademi University, Turku, Finland; ^3^Department of Psychological and Brain Sciences, University of Massachusetts Amherst, Amherst, MA, United States; ^4^Department of Psychiatry, Turku University Hospital and University of Turku, Turku, Finland; ^5^Department of Clinical Medicine, Turku University Hospital and University of Turku, Turku, Finland; ^6^Department of Psychology, University of Turku, Turku, Finland

**Keywords:** executive functioning, emotional availability, psychological distress, caregiving behavior, toddlerhood

## Abstract

Executive functioning (EF) is one of the building blocks in parental caregiving behavior, and contextual variables have been reported to moderate the link between EF and caregiving behavior. Although psychological distress due to various factors is prevalent during early parenthood and is negatively associated with adult EF, it is not known whether psychological distress influences the maternal EF/caregiving link. This study explored the association between maternal EF and caregiving behavior (more specifically, Emotional Availability/EA), and whether single and cumulative maternal psychological distress domains moderated the EF/EA association in a general population sample of 137 Finnish birth cohort mothers with 2.5-year-old children. EF was measured with a composite of five computerized Cogstate tasks, EA with the Emotional Availability Scales, and three psychological distress domains with self-report questionnaires (depression: EPDS, anxiety: SCL-90, insomnia: AIS). Better EF was significantly associated with more positive, sensitive caregiving, but this association was no longer significant when controlling for education level. Neither individual nor cumulative distress domains moderated the EF/EA association significantly, although the observed moderation effects were in the expected direction. These findings suggest that EF should be recognized alongside socioemotional factors as variables that are associated with parental caregiving behavior during toddlerhood. Furthermore, if the non-significant moderation results are replicated, they indicate that mothers in community samples are not at great risk for psychological distress that would compromise their capacity to utilize their EF while caring for their child. Further studies are needed to confirm these findings, as well as to examine these associations among fathers and in samples that have higher levels of chronic stressors. Studies with more diverse samples in terms of distress levels and EF performance would provide further insight into early childhood parenting and its risk factors.

## Introduction

Executive functioning (EF) enables goal-directed behavior like reasoning, problem solving, and planning. EF is associated with frontal–cingulate–parietal–subcortical networks, in which the prefrontal cortex plays a central role (Friedman and Miyake, 2017). The role of EF in maternal caregiving behavior is being increasingly studied (Bridgett et al., 2015; Crandall et al., 2015), but many central questions remain unanswered. To date, only two studies (Porreca et al., 2018; Harris et al., 2021) have explored the links between maternal EF and “Emotional Availability” (EA; Biringen, 2008). EA refers to a dyad’s capacity to share an emotionally healthy relationship, which is central for effective and supportive caregiving behavior. Rooted in attachment theory, EA broadens the perspective on dyadic interaction to include multiple features of parental functioning (i.e., sensitivity, structuring, non-intrusiveness and non-hostility). The EA framework also acknowledges the child’s contribution to the dyadic interaction and emphasizes the emotional feedback loop between the child and the parent (Biringen et al., 2014). As EA is known to be related to a wide variety of child outcomes (Saunders et al., 2015), a better understanding of how maternal EF is associated with mothers’ capacity to be emotionally available is needed.

Previous studies have indicated that the links between maternal EF and caregiving are complex. Among other things, contextual factors (i.e., socioeconomic variables, maternal sleep, and household chaos) have been reported to correlate with maternal EF, and to moderate its association with maternal caregiving (Deater-Deckard et al., 2012; Sturge-Apple et al., 2014; Chary et al., 2020). These findings highlight the importance of further studies on moderators of maternal EF/caregiving behavior links. To this end, in the current study we focused on various types of psychological distress, such as symptoms of depression, anxiety, and insomnia, because these are common in the general population during early parenthood (Goodman, 2004; Mindell et al., 2015; Goodman et al., 2016). They are also negatively associated with adult EF (Snyder et al., 2015; Ballesio et al., 2019). However, it is not known whether these distress domains influence the link between maternal EF and caregiving behavior during early parenthood.

### Maternal Executive Functions and Caregiving Behavior

The foundation of EF is thought to be made up of three interrelated core functions, i.e., working memory updating, set-shifting, and inhibitory control (Miyake et al., 2000). Researches have recently begun exploring the role of parental EF in caregiving behavior. Generally, lower maternal EF has been linked to harsher parenting and an increased risk of engaging in child maltreatment, while higher EF has been associated with sensitive, involved parenting (Bridgett et al., 2015; Crandall et al., 2015). When considering caregiving behavior from a neuropsychological perspective, it becomes clear why individual EF differences are related to caregiving quality. Children have constantly changing developmental needs, which present parents with a continuous stream of novel caregiving tasks that require flexible planning and problem-solving (Azar et al., 2008). Parents maintain and manipulate information in their working memory when planning child care, utilize their set-shifting ability to flexibly switch their attention across different situational demands in sometimes highly stimulating environments, and use inhibitory control to focus on their child’s needs and respond to them in a contingent and timely manner (Barrett and Fleming, 2011).

In the emerging research field of parental EF and caregiving behaviors, certain contextual factors appear to moderate the link between maternal EF and caregiving behavior. Deater-Deckard et al. (2012) reported that for mothers of 3-7-year-olds, child conduct problems were linked with harsh parenting, but only among mothers with lower EF in non-chaotic households. When studying mothers with 2.5-year-olds, Chary et al. (2020) found that both maternal sleep duration and sleep activity interacted with EF to predict the degree of harsh parenting. Among a socioeconomically diverse sample of mothers of 3-year-olds, Sturge-Apple et al. (2014) reported lower maternal working memory—a key aspect of EF—to be at risk for having dysfunctional child-centered responsibility attributions for child misbehavior, particularly under conditions of socioeconomic stress. Although these studies have utilized different measures of EF and caregiving behavior, they indicate that caregiving is linked to EF through a complex set of interactive processes involving contextual variables. Further studies are needed to elucidate the contextual factors involved, and how they may operate in various populations.

Clarifying the associations between maternal EF and caregiving behavior as well as moderating contextual factors is particularly important when examining early parenthood, because the first years of life are a sensitive period where psychosocial influences like caregiving behavior produce long-term effects on child development (Wachs et al., 2014). Also known as the “terrible twos,” toddlerhood is characterized by rapid cognitive, language and motor development and exploration of the physical world (Payne and Isaacs, 2017; Madigan et al., 2019), as well as by frequent emotional negative reactivity, like temper tantrums and noncompliance (Alink et al., 2006). Thus, toddlers require active parental caregiving, and the effects of parental EF capacity on caregiving behavior are likely to be particularly pronounced during this period. For this reason, we focus our current investigation on mothers of toddlers.

### Emotional Availability and Maternal Executive Functioning

The parent–child dyads’ EA is related to a wide range of child outcomes, such as emotion regulation, social competence, internalizing and externalizing problems, and language abilities (Saunders et al., 2015). Previous research has examined EA in relation to maternal variables like “mind mindedness,” sociodemographic variables, depression, and substance abuse (Biringen et al., 2014). In contrast, there is less knowledge about the role of maternal EF in EA. To the best of our knowledge, only two studies have explored this association. In a sample of 114 mostly highly educated Canadian mothers, Harris et al. (2021) performed repeated EA assessments as the children were one-and-a-half, three, and 5years old. A persistent, positive association between maternal EF (as measured with a composite score including inhibitory control and set-shifting) and EA trajectories was found, such that higher maternal EF statistically predicted increasing EA over time. Focusing on a sample of 29 Italian mothers with substance abuse disorder who had two-year-olds, Porreca et al. (2018) investigated how maternal EF and psychopathology were associated with EA. Better maternal EF was significantly associated with better EA. These findings suggest that maternal EF is one of the individual maternal factors that may be influencing EA and caregiving behavior, but more studies in a variety of samples are needed to confirm and extend these prior findings.

### Psychological Distress During Early Parenthood, Executive Functioning, and Emotional Availability

Besides being prevalent in early parenthood, the psychological distress domains depression, anxiety and insomnia are also negatively associated with adult EF. Postpartum depression, which has a global prevalence of 17.7% (Hahn-Holbrook et al., 2018), typically develops during the early postpartum period and often remits within a few months. However, some mothers’ symptoms are chronic. According to Goodman (2004), up to one third of the mothers who are depressed during the early postpartum period still suffer from depressive symptoms at 2years postpartum. Relatedly, approximately 8.5% of postpartum mothers experience one or more anxiety disorders (Goodman et al., 2016). In addition, sleep disturbances like nighttime awakenings and shorter sleep duration are common in early parenthood, with approximately 30% of mothers with children younger than 3years reporting that their daytime functioning is affected by their child’s sleep pattern (Mindell et al., 2015). When reviewing the associations between EF impairments and psychopathology, Snyder et al. (2015) comment that adults suffering from various psychopathologies perform worse on EF tasks than healthy controls. The same pattern is seen for adults diagnosed with insomnia (Ballesio et al., 2019). Sleep deprivation has been found to trigger brain activity changes that predict severity of impairment in working memory (Krause et al., 2017). Besides being negatively associated with adult EF, psychological distress has also been linked to maternal EA during early parenthood. Both maternal symptoms of depression and anxiety have been reported to be associated with lower maternal EA during the first postpartum year (see, e.g., Rossen et al., 2019; MacMillan et al., 2020; Frigerio and Nazzari, 2021). There are also findings from the FinnBrain Birth Cohort indicating that high and chronic maternal distress symptoms predict lower EA during early parenthood (Hakanen et al., 2019; Holmberg et al., 2021, Unpublished). Furthermore, maternal sleep fragmentation has been linked to lower maternal EA during the first year postpartum (Teti et al., 2016). To summarize, maternal symptoms of depression, anxiety, and insomnia are prevalent during early parenthood and are known to be negatively associated with both adult EF and with maternal EA during early parenthood. These domains of maternal psychological distress are therefore especially relevant to take into account when investigating the links between maternal EF and EA in early parenthood.

Whenever one is studying multiple domains of psychological distress, it is important to consider potential *cumulative* distress effects. Experiencing elevated symptoms in several domains of risk predicts more adverse consequences than experiencing symptoms within a single domain. In a cumulative distress model, combinations of different distress domains account for the variability in the outcome of interest (Evans et al., 2013). In a sample consisting partly of the same general population mothers of 2.5-year-old children as included in this study, we found that when studied as separate domains, symptoms of depression, anxiety, and insomnia were not significantly associated with EF. However, a negative association with EF was found when the overall number of clinically elevated distress domains was examined, so that mothers reporting several concurrent clinically elevated distress domains tended to have lower EF (Nordenswan et al., submitted). This finding underscores the relevance of considering cumulative effects when studying these multiple domains of risk as moderators of the link between maternal EF and caregiving behavior.

### The Current Study

There is ample evidence that maternal EF covaries with and probably influences parenting behavior (Crandall et al., 2015). Also, contextual variables have been found to moderate the links between maternal EF and caregiving (Deater-Deckard et al., 2012; Sturge-Apple et al., 2014; Chary et al., 2020). Although psychological distress is common during early parenthood (Goodman, 2004; Mindell et al., 2015; Goodman et al., 2016) and is negatively associated with adult EF (Snyder et al., 2015; Ballesio et al., 2019), existing studies have not yet explored whether the link between maternal EF and caregiving behavior varies as a function of the level of psychological distress during early parenthood. Our study addresses this gap in the literature. Using a sample of general population mothers with 2.5-year-old children, which was drawn from a Finnish birth cohort study that explores child development and parenting (Karlsson et al., 2018), we examined whether maternal EF was associated with caregiving behavior (i.e., EA; Biringen, 2008). Furthermore, we explored whether three domains of maternal psychological distress (i.e., symptoms of depression, anxiety, and insomnia) moderated this association.

In line with the previous research presented above, our first hypothesis was that better maternal EF would be related to higher maternal EA. Second, we expected this association to be moderated by maternal psychological distress—specifically, that the strength of the anticipated link between maternal EF and EA would be stronger for mothers with lower symptom levels, but weaker for mothers with higher symptom levels. We also expected to find cumulative effects, so that the moderation effect would be more pronounced for cumulative psychological distress scores compared to single psychological distress domains.

## Materials And Methods

### Participants and Procedure

Participants were recruited from the FinnBrain Birth Cohort (*N*=4,000 families; Karlsson et al., 2018). The main cohort was recruited in Southwest Finland between 2011 and 2015. Families joined the study during gestational week 12, when attending free-of-charge pregnancy ultrasound scans at maternal welfare clinics. A normal ultrasound screening result and sufficient knowledge of Finnish or Swedish were required for participation (Karlsson et al., 2018).

**Figure 1:**
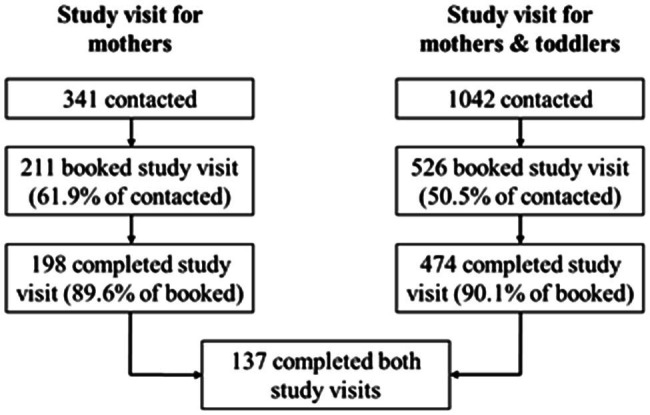
Participant recruitment to the study visits. Maternal EF and psychological distress were measured during the study visit for only mothers, maternal caregiving behavior was assessed during the joint study visit for mothers and toddlers. Both visits were conducted when the child was 2.5years old. Of the 341 mothers who were informed about the study visit for only mothers, the mothers who completed the study visit were significantly higher educated than the non-participating mothers, *X*^2^ (2, *N*=341)=8.48, *p*=0.01. The participating and non-participating mothers did not differ significantly in terms of age [*t*(339)=1.74, *p*=0.08] or number of children [*X*^2^ (4, *N*=341)=4.68, *p*=0.32]. Of the 1,042 mothers who were informed about the joint study visit for mothers and toddlers, the mothers who completed the study visit were significantly older than the non-participating mothers, *t*(1036)=−4.99, *p*<0.001. The participating mothers were higher educated than the non-participating mothers [*X*^2^ (2, *N*=1,042)=25.07, *p*<0.001], but the participating and non-participating mothers did not differ significantly in terms of number of children [*X*^2^ (5, *N*=1,042)=4.78, *p*=0.44]. The 137 mothers who participated in both study visits were significantly older [*t*(3806)=2.39, *p*=0.02] and more highly educated [*X*^2^ (2, *N*=3,808)=13.53, *p*=0.00] than the rest of the birth cohort’s mothers.

The current study’s participants (*N*=137 mothers) took part in a broader sub-study within FinnBrain, which explores child self-regulation development and parenting. Within this sub-study, mothers from the main FinnBrain birth cohort were from 2012 to 2013 randomly selected for recruitment to a study visit examining parental cognitive functioning, including EF tasks among other measures. Exclusion criteria were self-reported psychiatric or neurologic illness and insufficient Finnish language skills. The parental cognition sub-study’s first study visit was conducted during pregnancy. Mothers who had attended the first visit (*N*=274) were invited back for follow-up visits at 1year and 2.5years after delivery. At the recruitment of participants to the 2.5-year study visit, the recruitment list was enriched with mothers whose children had participated in an own study visit assessing child self-regulation development. In total, 198 mothers completed the study visit assessing parental cognitive functioning at 2.5years postpartum, during which computerized EF measures and questionnaires assessing symptoms of depression, anxiety, and sleep disturbance were completed along with other tasks. For 137 of these mothers, data on caregiving behavior had been collected at a separate study visit, which they had attended together with their 2.5-year-old toddler. During that visit, a 15–20min long free-play situation was completed and video recorded. Age-appropriate toys were provided on a comfortable floor carpet during the free-play situation. Both study visits were conducted by graduate students in quiet examination rooms. Participant recruitment is described in more detail in Figure 1. The Joint Ethics Committee of Turku University Hospital and University of Turku gave ethical approval for this study. Written informed consent was obtained from the mothers before participation.

During the data collection of the main variables of interest for this study (i.e., at 2.5years postpartum), the participants had a mean age of 34.30years (*SD*=4.83years, range=21.81–44.92years). At this time, more than half of the mothers (62.5%) were primiparous, 22.7% had two children, 14.1% had three children, and 0.8% had four children. At 2years postpartum, approximately two thirds (73.7%) of this study’s participants filled out a couple relationship questionnaire, indicating that they were in a couple relationship at the time, while 26.3% did not. Although data on marital status were not collected at 2.5years postpartum, this offers a rough estimation of the number of participants in a couple relationship. Information about participant income level, education level, and occupation was gathered approximately 3years prior to the collection of this study’s main variables of interest, when the pregnant mothers were recruited to the main FinnBrain cohort. At this time, almost half of the mothers (46.3%) had a university level education, 29.4% had a polytechnics education, while 24.3% had a high school/vocational education (<12years). A majority of the participants (78.7%) were employed, while few were unemployed (2.9%), students (6.6%), stay-at-home mothers (7.4%), or otherwise occupied (4.4%). After taxes, 29.6% of the participants had a total monthly income of 1,500€ or less, 57.8% had an income between 1,501€ and 2,500€, 10.4% had an income between 2,501 and 3,500€, and 2.2% had an income over 3,500€.

### Measures

#### Executive Functions

EF was measured with five Cogstate tasks, which were combined into a composite. Cogstate tasks are computerized adaptations of standard neuropsychological test (Pietrzak et al., 2009). It is preferable to base EF assessment on multiple tasks, as any EF task engages both general (EF) and task-specific cognitive processes (Friedman and Miyake, 2017). The participants completed a 12-task Cogstate test battery, from which five tasks thought to tap into EF were included in this study. The task outcome variables thought to best capture EF-related variance were utilized, in line with a previous factor analytic study (Nordenswan et al., 2020). As EF is engaged especially during the early stages of cognitive task performance, the first test round’s result was selected instead of all rounds’ summative score (for any tasks that had multiple test rounds). For the Continuous Paired Associate Learning Test and for the Groton Maze Learning Test, the outcome variables were reversed, so that a higher value equated with a better EF. The five task scores were standardized, calculated into an EF average score, and re-standardized. The tasks included in the EF composite are described briefly below, and in more detail in Nordenswan et al. (2020).

##### The International Shopping List Test

In this verbal list learning task, a shopping list of 12 items is read out loud, and the participant is instructed to recall the items. The International Shopping List (ISL) includes three rounds, during which the same procedure is repeated. We utilized the number of correct responses from the first round.

##### The Groton Maze Learning Test

In this hidden maze task, the participant is first taught the task rules in a small practice grid. During the actual task, the participant guesses a 28-step pathway, which is hidden among 100 possible locations in a 10×10 grid of tiles. The Groton Maze Learning Test (GML) includes five test rounds, during which the same pathway is uncovered. The number of errors from the first test round (i.e., the round after the first learning trial) was used.

##### The Set-Shifting Test

(SETS) is similar to the Wisconsin Card Sorting Test. The participant is instructed to guess whether a playing card contains a target stimulus, i.e., a color or number. A correct response is required before the next card is shown, and thus, the participant is taught which card dimension is correct. After a while, the correct dimension changes, and the participant must learn the new rule to proceed. After 120 correct responses, the task is terminated. We used the arcsine transformation of the square root of the proportion of correct responses.

##### The Two Back Test

(TWOB) measures working memory. In this task, a playing card is shown at the center of the screen, and the participant is to decide whether it is the same card that was presented two rounds previously. Thirty-two correct responses are required for the task to terminate. The arcsine transformation of the square root of the proportion of correct responses was used.

##### The Continuous Paired Associate Learning Test

(CPAL) assesses the capacity to encode associations between simple patterns and spatial locations, so that exposure to one aspect of the information prompts the recall of the other. First, the participant is taught the location of eight figures that are hidden behind circles on the screen. As the figures are then shown at the screen’s center, the participant is to remember behind which circle the figure is hidden. The same procedure is repeated during six test rounds. We utilized the number of errors from the first test round (i.e., the round after the first learning trial).

#### Caregiving Behavior

The Emotional Availability Scales (EA; Biringen, 2008) were used to code maternal caregiving behavior during the free-play situations. Emotional availability refers to a dyad’s ability to share an emotionally healthy relationship (Biringen et al., 2014). The EA scales have been extensively studied and are seen as a sensitive and valid measure of relational dyadic affective quality. They are associated with both child–parent attachment, and child socioemotional adaptation (Biringen et al., 2014). Four EA dimensions describe separable aspects of parental caregiving behavior. Sensitivity refers to having a positive and authentic emotional presence, while appropriately reading and responding to the child’s emotional cues. Structuring describes the capacity to guide and mentor the child’s pursuits, while strengthening the child’s sense of autonomy. Non-intrusiveness refers to not interfering with, over-stimulating, over-directing, or overprotecting the child. Non-hostility describes the absence of hostile, threatening, or frightening behavior. The EA dimensions are scored from 1 to 7 points, on a 14-point Likert scale. For all dimensions, scores from 1 to 2 are considered highly problematic, scores from 2.5 to 3.5 indicate detachment in the relationship, scores from 4 to 5 are viewed as somewhat problematic and indicate complicated EA, whereas scores from 5.5 to 7 are indicative of healthy EA in the relationship (Biringen and Easterbrooks, 2012). The coding was done by three coders, who received training and a certificate of reliability from the developer of the EA scales (Biringen). Interrater reliability was assessed for 10% of the play episodes. The intraclass correlation coefficient for sensitivity ranged from 0.83 to 0.91, for structuring from 0.84 to 0.91, for non-intrusiveness from 0.84 to 0.90, and for non-hostility from 0.70 to 0.85. Coding differences were negotiated between the coders. To reduce the amount of parameters being estimated in analyses, the four maternal EA dimensions were averaged, and then standardized (composite EA scores have been used also elsewhere, see, e.g., Salo et al., 2020). Higher values on this caregiving composite describe better emotional availability, i.e., more sensitivity/structuring and less intrusiveness/hostility.

#### Psychological Distress

##### Depression Symptoms

We used the Edinburgh Postnatal Depression Scale (EPDS; Cox et al., 1987) to measure depression symptoms. This self-report questionnaire consists of 10 items that assesses depression symptoms experienced during the past 2weeks using a 4-point Likert scale. The EPDS has been extensively studied and is seen as a valid measure of postnatal depression (Smith-Nielsen et al., 2018). We utilized the EPDS total score (higher value=more symptoms of depression). A cutoff score of 11 or more indicates depression (Smith-Nielsen et al., 2018).

##### Anxiety Symptoms

The anxiety subscale from the Symptom Checklist 90 questionnaire (SCL-90; Derogatis et al., 1973) was used to assess symptoms of anxiety. The SCL-90 anxiety subscale consists of 10 self-report items, which measure anxiety symptoms experienced during the past month, using a 5-point Likert scale. One participant had one missing value on the SCL-90 anxiety subscale, which was imputed with the other items’ mean value. We utilized the SCL-90 anxiety subscale total score (higher value=more symptoms of anxiety). A cutoff level of 7.5 points or more indicates a clinically elevated anxiety level (Schmitz et al., 2000).

##### Insomnia Symptoms

We used the Athens Insomnia scale (AIS; Soldatos et al., 2000) to assess insomnia symptoms. This self-report questionnaire consists of eight items and is designed for quantification of sleep difficulty based on the International Classification of Diseases (ICD-10) criteria. The AIS has been found to have sound psychometric properties (Soldatos et al., 2003). We used the total AIS score (higher value=more symptoms of insomnia). A cutoff score of 6 or higher points to insomnia (Soldatos et al., 2003).

### Analytic Approach

All analyses were performed with SPSS (version 26). All variables were evaluated for normality, and the Cogstate completion pass rates and integrity pass rates were calculated. Mean values and standard deviations were calculated for the Cogstate tasks, the EA scales and the symptom questionnaires. For the Cogstate tasks with available normative data (i.e., TWOB, GML, CPAL, and ISL), results were compared with unpublished normative data for healthy adults (Cogstate, 2014, Unpublished). Some mothers had undergone a previous Cogstate testing. Practice effects were controlled for by comparing the first-time participants’ results (*n*=103) with re-tested participants’ results (*n*=34) using the Mann–Whitney *U* test. The EPDS, SCL-90 and AIS scores were compared with recommended cutoff scores. Bivariate correlations between the covariates, the independent variable, the dependent variable, and the moderator variables were calculated.

The EF/caregiving association, as well as the moderating effect of psychological distress (i.e., depression, anxiety, and insomnia) on this association, were explored with four separate hierarchical multiple regression analyses. The regression models included separate tests of each of the three distress variables, as well as a cumulative distress composite variable described below. To examine the robustness of the EF/caregiving relationship and the possible moderator effects, the hierarchical regression models were run without and then with education level as a covariate in Step 1. We wanted to employ education level in the analyses due to its significant association with caregiving behavior (Table 1).

**Table 1:** Bivariate correlations between the study variables.

S. No	Variables	1	2	3	4	5	6	7	8	9
**1.**	**EF composite** ^**a**^	1								
**2.**	**Caregiving composite** ^**a**^	0.17^*^	1							
**3.**	**Depression symptoms** ^**b**^	−0.01	−0.01	1						
**4.**	**Anxiety symptoms** ^**b**^	0.02	0.01	0.79^**^	1					
**5.**	**Insomnia symptoms** ^**b**^	−0.11	−0.04	0.52^**^	0.40^**^	1				
**6.**	**Distress composite** ^**b**^	−0.04	−0.02	0.91^**^	0.87^**^	0.76^**^	1			
**7.**	**Age**	−0.05	0.03	−0.02	−0.13	0.10	−0.02	1		
**8.**	**Number of children**	0.10	0.06	−0.05	−0.03	−0.14	−0.02	0.36^**^	1	
**9.**	**Education level** ^**a**^	0.23^*^	0.17^*^	0.02	−0.07	0.08	0.02	0.36^**^	0.11	1

* Correlation is significant at the 0.05 level.

** Correlation is significant at the 0.01 level.

a Higher score=more advantageous.

b Lower score=more advantageous.

Pearson correlations were calculated for all variables except for education level, for which Spearman correlations were calculated. EF composite=standardized mean value of five Cogstate executive functioning tasks. Caregiving composite=standardized mean value of four maternal emotional availability dimensions. Depression symptoms=Edinburgh Postnatal Depression Scale. Anxiety symptoms=Symptom Checklist-90 anxiety subscale. Insomnia symptoms=Athens Insomnia Scale.

The three psychological distress domains (i.e., depression, anxiety, and insomnia) were added as continuous variables to three separate models, allowing for an examination of their individual influences on the EF/caregiving association. The EF composite was added to Step 1 of all three models. In Step 2, we added one distress domain per model (Model 1: EPDS, Model 2: SCL-90, Model 3: AIS). In Step 3, we added the interaction term between the distress domains and EF (Model 1: EPDS x EF, Model 2: SCL-90 x EF, Model 3: AIS x EF). Models 1–3 were then re-run, with education level controlled for in Step 1.

Next, we combined the three psychological distress domains into a composite, in order to detect a potential cumulative effect on the EF/caregiving association. The EPDS, SCL-90, and AIS variables were standardized, averaged, and then re-standardized. In this, the fourth regression model, the EF composite was again added in Step 1. In Step 2, we added the EPDS/SCL-90/AIS composite. In Step 3, we added the interaction term between the psychological distress composite and EF (EPDS/SCL-90/AIS x EF). Finally, the fourth regression model was re-run with education level controlled for in Step 1.

## Results

### Descriptive Statistics

The means, standard deviations, and ranges for the study variables are presented in Table 2. Seven participants’ TWOB results were excluded due to an insufficient pass rate, and two participants’ SETS results were excluded, as they were incomplete. Besides this, all other Cogstate tasks’ integrity pass and completion pass rates were 100%. The TWOB and GML results were within the normal range (±1SD) of Cogstate normative data for the age groups 18-34yrs. and 35-49yrs. The participants’ ISL results were slightly better than for both normative age groups. More errors were made on the CPAL than expected based on the norms; however, the CPAL normative sample size is very small (18–34years *N*=62, 35–49years *N*=9) and should thus be referred to with caution. Regarding potential learning effects, mothers tested with Cogstate for the first time vs. the re-tested mothers did not have significantly different results (separate *U*-tests for five Cogstate tasks, *p*=0.15–0.79). As expected in a general population sample, the four EA dimensions showed that the mothers were exhibiting mostly positive, emotionally available caregiving behavior. The majority of the free-play situations were coded as healthy emotional availability (sensitivity: 49.6%, structuring 50.4%, non-intrusiveness: 67.9%, non-hostility: 91.2%), some were coded as somewhat problematic (sensitivity: 37.2%, structuring 38.7%, non-intrusiveness: 24.8%, non-hostility: 8.0%), few were coded as detached in the relationship (sensitivity: 12.4%, structuring 10.2%, non-intrusiveness: 7.3%, non-hostility: 0.7%), and almost none were coded as highly problematic (sensitivity: 0.7%, structuring 0.7%, non-intrusiveness: 0.0%, non-hostility: 0.0%).

**Table 2:** Mean values, standard deviations and ranges for study variables.

Variable	Mean	*SD*	Range
**Cogstate**			
Two Back Test	1.31	0.12	1.03–1.57
Set-Shifting Test	1.19	0.11	0.92–1.33
Groton Maze Learning Test	8.33	3.42	1–19
Continuous Paired Associate Learning Test	12.91	8.70	0–40
International Shopping List Test	8.05	1.62	4–12
**Emotional availability scales**			
Sensitivity	5.11	1.15	2–7
Structuring	5.25	1.23	2–7
Non-intrusiveness	5.92	1.28	3–7
Non-hostility	6.49	0.79	3–7
EA dimensions, mean value	5.69	0.97	2.75–7.00
**Questionnaires**			
**Edinburgh Postnatal Depression Scale**	4.00	4.02	0–18
**Symptom Checklist 90, anxiety subscale**	3.42	4.32	0–19
**Athens Insomnia Scale**	5.89	3.42	0–18

The outcome measures are described in the Materials and Methods, and Measures section.

Depression and anxiety symptoms were similarly distributed in the study sample. Many participants reported no symptoms (EPDS: 20.4%, SCL-90: 31.4%). A larger proportion reported subclinical symptoms (EPDS: 68.6%, SCL-90: 54.0%), while a small group reported clinically elevated symptom levels (EPDS: 10.9%, SCL-90: 14.6%). In contrast, insomnia symptoms were more prevalent in the sample. Only 4.4% reported no insomnia symptoms, 42.3% reported subclinical insomnia symptoms, and 53.3% reported clinically elevated insomnia symptoms.

### Correlation Results

The bivariate correlations between the study variables and covariates are presented in Table 1. As expected, the “better EF/better caregiving” association was significant, but had a small effect size. The psychological distress domains did not correlate significantly with either EF, or with caregiving. However, the distress domains all covaried significantly with each other. The depression/anxiety association had a large effect size, while the insomnia/depression and the insomnia/anxiety associations had a medium effect size. Of the potential control variables (i.e., participant age, number of children, and education level), only education level correlated significantly with caregiving. Thus, we chose to only include education level as a control variable in the subsequent hierarchical multiple regression analyses.

### Regression Results

#### The Association Between EF and Caregiving

As can be seen in the first analysis steps in Tables 3 and 4, better EF was significantly associated with better caregiving when education was not included as a control variable (∆*R*^2^=0.03, *p*=0.04); mom EF accounted for 3% of the variation in caregiving behavior. When education level was added as a control variable to Step 1 (Tables 5 and 6), the EF/caregiving association weakened slightly and was no longer statistically significant (∆*R*^2^=0.02, *p*=0.12). In the regression models that included education as a control variable in Step 1 (Tables 5 and 6), education level accounted for 3% of the variation in caregiving behavior (∆*R*^2^=0.03, *p*=0.04).

**Table 3:** Single distress domains’ moderation of EF/caregiving association, excluding covariate.

	*R* ^2^	*R*^2^∆	*F*∆	*F*∆	*B*	*β*	*t*	*B*	*B*, 95.0% CI	*sr* ^2^
								*p*		
				*p*						
**Depression symptoms**										
**Step 1**: EF	0.03	0.03	4.19	0.04	0.17	0.17	2.05	0.04	0.01/0.34	0.03
**Step 2**: EPDS	0.03	0.00	0.00	0.94	−0.01	−0.01	−0.08	0.94	−0.18/0.16	0.00
**Step 3**: EPDS X EF	0.04	0.01	1.53	0.22	−0.10	−0.11	−1.24	0.22	−0.27/0.06	0.01
**Anxiety symptoms**										
**Step 1**: EF	0.03	0.03	4.19	0.04	0.17	0.17	2.05	0.04	0.01/0.34	0.03
**Step 2**: SCL-90	0.03	0.00	0.00	0.99	0.00	0.00	0.01	0.99	−0.17/0.17	0.00
**Step 3**: SCL-90 X EF	0.03	0.00	0.04	0.84	−0.02	−0.02	−0.21	0.84	−0.19/0.16	0.00
**Insomnia symptoms**										
**Step 1**: EF	0.03	0.03	4.19	0.04	0.17	0.17	2.05	0.04	0.01/0.34	0.03
**Step 2**: AIS	0.03	0.00	0.08	0.78	−0.03	−0.03	−0.29	0.78	−0.19/0.15	0.00
**Step 3**: AIS X EF	0.05	0.01	1.95	0.17	−0.12	−0.12	−1.40	0.17	−0.28/0.05	0.01

EF, executive functioning, five-task-Cogstate-composite; EPDS, Edinburgh Postnatal Depression Scale; SCL-90, Symptom Checklist-90 anxiety subscale; AIS, Athens Insomnia Scale.

**Table 4:** Distress composite’s moderation of EF/caregiving association, excluding covariate.

	*R* ^2^	*R*^2^∆	*F*∆	*F*∆	*B*	*β*	*t*	*B*	*B*, 95.0% CI	*sr* ^2^
								*p*		
				*p*						
**Step 1**: EF	0.03	0.03	4.19	0.04	0.17	0.17	2.05	0.04	0.01/0.34	0.03
**Step 2**: Symptoms	0.03	0.00	0.02	0.89	−0.01	−0.01	−0.14	0.89	−0.18/0.16	0.00
**Step 3**: Symptoms X EF	0.04	0.01	1.21	0.27	−0.09	−0.09	−1.10	0.27	−0.26/0.07	0.01

EF, executive functioning, five-task-Cogstate-composite. Symptoms=a cumulative composite score, combines concurrent symptoms of depression (measured with the Edinburgh Postnatal Depression Scale), anxiety (measured with the Symptom Checklist-90 anxiety subscale), and Insomnia (measured with the Athens Insomnia Scale).

**Table 5:** Single distress domains’ moderation of EF/caregiving association, including covariate.

	*R* ^2^	*R*^2^∆	*F*∆	*F*∆	*B*	*β*	*t*	*B*	*B*, 95.0% CI	*sr* ^2^
				*p*				*p*		
**Depression symptoms**										
**Step 1**: Education level	0.03	0.03	4.38	0.04	0.22	0.18	2.09	0.04	0.01/0.43	0.03
**Step 2**: EF	0.05	0.02	2.48	0.12	0.14	0.14	1.57	0.12	−0.04/0.31	0.02
**Step 3**: EPDS	0.05	0.00	0.00	0.97	0.00	0.01	0.04	0.97	−0.17/0.17	0.00
**Step 4**: EPDS X EF	0.06	0.01	0.92	0.34	−0.08	−0.08	−0.96	0.34	−0.25/0.09	0.01
**Anxiety symptoms**										
**Step 1**: Education level	0.03	0.03	4.38	0.04	0.22	0.18	2.09	0.04	0.01/0.43	0.03
**Step 2**: EF	0.05	0.02	2.48	0.12	0.14	0.14	1.57	0.12	−0.04/0.31	0.02
**Step 3**: SCL-90	0.05	0.00	0.09	0.76	0.03	0.03	0.30	0.76	−0.14/0.20	0.00
**Step 4**: SCL-90 X EF	0.05	0.00	0.00	0.97	−0.00	−0.00	−0.04	0.97	−0.18/0.17	0.00
**Insomnia symptoms**										
**Step 1**: Education level	0.03	0.03	4.38	0.04	0.22	0.18	2.09	0.04	0.01/0.43	0.03
**Step 2**: EF	0.05	0.02	2.48	0.12	0.14	0.14	1.57	0.12	−0.04/0.31	0.02
**Step 3**: AIS	0.05	0.00	0.25	0.62	−0.04	−0.04	−0.50	0.62	−0.21/0.13	0.00
**Step 4**: AIS X EF	0.06	0.01	1.67	0.20	−0.11	−0.11	−1.29	0.20	−0.27/0.06	0.01

EF, executive functioning, five-task-Cogstate-composite; EPDS, Edinburgh Postnatal Depression Scale; SCL-90, Symptom Checklist-90 anxiety subscale; AIS, Athens Insomnia Scale.

**Table 6:** Distress composite’s moderation of EF/caregiving association, including covariate.

	*R* ^2^	*R*^2^∆	*F*∆	*F*∆	*B*	*β*	*t*	*B*	*B*, 95.0% CI	*sr* ^2^
				*p*				*p*		
**Step 1**: Education level	0.03	0.03	4.38	0.04	0.22	0.18	2.09	0.04	0.01/0.43	0.03
**Step 2**: EF	0.05	0.02	2.48	0.12	0.14	0.14	1.57	0.12	−0.04/0.31	0.02
**Step 3**: Symptoms	0.05	0.00	0.00	0.95	−0.01	−0.01	−0.06	0.95	−0.17/0.16	0.00
**Step 4**: Symptoms X EF	0.06	0.01	0.75	0.39	−0.07	−0.07	−0.86	0.39	−0.24/0.09	0.01

EF, executive functioning, five-task-Cogstate-composite. Symptoms = a cumulative composite score, combines concurrent symptoms of depression (measured with the Postnatal Depression Scale), anxiety (measured with the Symptom Checklist-90 anxiety subscale), and insomnia (measured with the Athens Insomnia Scale).

#### The Moderating Role of Single Psychological Distress Domains

The results of the three hierarchical multiple regression analyses to which the psychological distress domains were added individually are first presented in Table 3 without education as a control variable, and then with education included in Table 5. Symptoms of depression, anxiety, and insomnia were not significantly associated with caregiving behavior, whether education level was controlled (EPDS: ∆*R*^2^=0.00, *p*=0.97; SCL-90: ∆*R*^2^=0.00, *p*=0.76; AIS: ∆*R*^2^=0.00, *p*=0.62) or not (EPDS: ∆*R*^2^=0.00, *p*=0.94; SCL-90: ∆*R*^2^=0.00, *p*=0.99; AIS: ∆*R*^2^=0.00, *p*=0.78). Contrary to our expectations, the interaction terms between the distress domains and EF were not significantly associated with caregiving behavior, whether education level was controlled (EPDS X EF: ∆*R*^2^=0.01, *p*=0.34; SCL-90 X EF: ∆*R*^2^=0.00, *p*=0.97; AIS X EF: ∆*R*^2^=0.01, *p*=0.20) or not (EPDS X EF: ∆*R*^2^=0.01, *p*=0.22; SCL-90 X EF: ∆*R*^2^=0.00, *p*=0.84; AIS X EF: ∆*R*^2^=0.01, *p*=0.17). When analyzed as individual distress domains, symptoms of depression, anxiety, and insomnia did not appear to have a significant moderation effect on the association between EF and caregiving behavior.

#### The Moderating Role of Cumulative Psychological Distress

The results of the fourth regression analysis using the cumulative psychological distress composite are first presented in Table 4 without the education covariate, and then including education as a covariate in Table 6. The distress composite was not significantly associated with caregiving behavior, whether education level was controlled (∆*R*^2^=0.00, *p*=0.95) or not (∆*R*^2^=0.00, *p*=0.89). Contrary to our expectations, the interaction term between the distress composite and EF was not significantly associated with caregiving behavior, whether education level was controlled (∆*R*^2^=0.01, *p*=0.39) or not (∆*R*^2^=0.01, *p*=0.27). When combined into a composite, depression, anxiety, and insomnia symptoms did not moderate the EF/caregiving association.

## Discussion

We examined the associations between maternal EF and caregiving behavior—operationalized as EA—when the child was 2.5years old in a general population sample drawn from a Finnish birth cohort (Karlsson et al., 2018). Furthermore, we explored whether single and cumulative psychological distress domains (i.e., depression, anxiety, and insomnia) moderated the EF/caregiving association. Firstly, higher maternal EF was expected to be associated with better caregiving behavior. Secondly, the psychological distress domains were hypothesized to moderate this association, so that it would be stronger for mothers with lower symptom levels. The moderator effect was expected to be more pronounced for cumulative psychological distress scores in comparison with single distress domains. Our hypotheses were partly supported. Better maternal EF was significantly associated with better caregiving behavior, but when education level was controlled for this association weakened and was no longer significant (*p*=0.12). None of the individual distress domains, nor a cumulative distress composite, moderated the EF/caregiving association, although the observed moderation effects were in the expected direction.

The association between higher maternal EF and better EA found in this study is in line with previous literature, which generally has linked better maternal EF with more involved and sensitive parenting (Bridgett et al., 2015; Crandall et al., 2015). To the best of our knowledge, only one previous study has examined the associations between maternal EF and specifically EA among general population mothers during early parenthood. In that study (Harris et al., 2021), the authors found an EF composite of inhibitory control and set-shifting capacity to predict better maternal EA. Our study supports these findings and complements them by offering insight into how slightly different EF aspects are related to maternal EA when the child is 2.5years old. However, the association found in this study between maternal EF and caregiving was weak and did not remain statistically significant after controlling for education level. This weak association is understandable, as the origins of parenting is a complex phenomenon that involves multiple factors, like family socioeconomic status, maternal mental health, and child characteristics (Biringen et al., 2014). In other words, EF is only one variable among many others that affect caregiving behavior. The weakening effect of including education level as a covariate is also logical, considering that EF and education level are closely intertwined (Deary and Johnson, 2010). The maternal EF/EA association we found is very similar to the association reported by Harris et al. (2021)—in their sample, the EF/EA correlation was 0.23^**^, while it was 0.17^*^ in our sample. However, in Harris et al. (2021), the effect of maternal EF on EA remained significant when controlling for a family socioeconomic status composite that included maternal education level (as well as household income). It is possible that this differing result is due to the combination of maternal education level (which is likely to covary with maternal EF) with a variable that might not covary with maternal EF, i.e., household income, which can be largely influenced by a spouse’s employment. Notably, the Harris et al. (2021) family socioeconomic status composite did not correlate significantly with maternal EF (−0.01), but maternal education level did correlate significantly with EF in our sample (0.21^*^).

The single psychological distress domains, and the averaged cumulative distress composite, did not moderate the EF/caregiving association. However, the direction of the observed interaction effects was in line with our hypotheses, suggesting that higher levels of psychological distress could weaken the association between better EF and better EA. It is possible that the non-significant results are due to the relatively small sample size and to lower power to detect interaction effects (compared to power to detect “main effects”). The lack of a significant interaction also could arise from having too little variance and low numbers of mothers with elevated psychiatric symptoms. If the non-significant moderation result is replicated in larger samples, this would indicate that mothers in general population or community samples are not at great risk for psychological distress that would compromise their capacity to utilize their EF while caring for their child.

Importantly, this null finding should not be generalized to mothers who face higher levels of chronic stressors, as psychological distress could affect the EF/caregiving association in other samples with a broader range of symptom levels and stress exposures. Our general population sample had fairly low levels of stress exposure and symptoms; as a group, the participants reported few symptoms of depression/anxiety, displayed healthy EA levels when playing with their child, had a normative level of verbal intelligence, and were fairly highly educated. In a more psychologically distressed sample (i.e., mothers with substance abuse), Porreca et al. (2018) found maternal EF to account for 25.6% of the variation in maternal EA. Their reported effect size is stronger than the effect found in the current study (in which EF accounted for only 3% of the variation in maternal EA), suggesting that sample characteristics may affect the strength of the association that is found between maternal EF and EA.

Although in the current study we did not focus on how maternal psychological distress is associated with EA, the link between these variables in our sample warrants attention, as this is a widely studied research topic elsewhere (see, e.g., Rossen et al., 2019; MacMillan et al., 2020; Frigerio and Nazzari, 2021). In our regression models, symptoms of depression, anxiety, and insomnia were not significantly associated with maternal EA. This was true whether we examined the symptom domains separately or as part of a combined cumulative composite, or whether or not we included education as a covariate. Our findings are in line with previous research indicating that maternal psychological distress is differently associated with maternal EA depending on the timing, severity, and chronicity of the distress symptoms, as well as on the presence of other contextual risk factors. Korja and McMahon (2021) recently found that maternal prenatal, but not postnatal, symptoms of depression and anxiety were associated with maternal EA at 6months postpartum. Similarly, MacMillan et al. (2020) reported that maternal prenatal depression symptoms have a small effect on maternal EA at 6months postpartum, while there was no effect of a postnatal depression diagnosis on maternal EA. In a consecutive study, MacMillan et al. (2021) found the association between maternal depressive symptoms during early pregnancy and EA at 6 months postpartum to be moderated by maternal postpartum perceptions of partner and family social support. This indicates that the effect of on EA might in some populations be evident only when other contextual risk factors are also present. Examining partly the same mothers from the FinnBrain Birth Cohort as the mothers included in this study’s sample, Hakanen et al. (2019) found only a couple associations with small effect sizes between maternal pre- and postnatal anxiety/depression (repeatedly assessed three times during pregnancy and twice postpartum) and the four dimensions of maternal EA at 8months postpartum. Also utilizing partly the same study sample, Holmberg et al. (2021, Unpublished) examined longitudinal maternal pre- and postnatal depression/anxiety symptom patterns in relation to sensitivity, a dimension of maternal EA. Chronically high levels of psychological distress from pregnancy to toddlerhood was associated with maternal sensitivity at 2.5years after delivery. This suggests that chronic distress could be differently associated with EA than more transient distress symptoms. In light of these findings, it is understandable that maternal psychological distress and EA were not associated in our sample, as distress/EA associations can differ depending on the timing of the symptom measurements, our distress measurements did not evaluate symptom chronicity, and our highly educated general population participants are not at high risk for other contextual adversities that could moderate the distress/EA association.

### Caveats, Limitations, and Strengths

This study has certain caveats, limitations, and strengths to take into account. Most importantly, as our sample was not focused on recruiting participants with clinically-relevant levels of psychiatric symptoms, the results cannot be generalized to mothers experiencing high levels of psychosocial stressors. In addition, the study design was correlational, therefore making it not possible to infer causality in the associations we examined.

Another limitation is that EF capacity may be utilized differently in a structured laboratory environment compared to real-life situations. This raises questions concerning how accurately the EF measurements describe the participants’ capacities to use their EF when caring for their child day to day. However, laboratory tasks are considered a “gold standard” for EF measurement and have better predictive validity than questionnaires.

A related strength is that we used a highly reliable composite score based on several EF tasks that minimizes random measurement error. However, the five EF tasks in our composite primarily measured working memory and set-shifting but not inhibitory control—a limitation of our study. Also, it should be noted that the EF tasks we used incorporate notable elements of learning, and could be more broadly defined as “EF/learning tasks” (Nordenswan et al., 2020). Finally, self-report questionnaires were used to assess psychological distress. Potential reporting biases are thus possible. However, the questionnaires we used capture a central aspect of ecological validity—healthcare providers assessing maternal psychological distress during early parenthood often use the same or very similar measures to those that we used.

### Conclusion

Our results showed that for a general population sample of Finnish mothers of 2.5-year-olds, better EF is weakly associated with better emotional availability. Psychological distress domains that are common during early parenthood (i.e., depression, anxiety, and insomnia) did not moderate the EF/caregiving association, although the observed moderation effects were in the expected direction. These findings suggest that, e.g., in the context of parenting interventions, EF should be recognized alongside socioemotional factors as variables that are associated with parental caregiving behavior in toddlerhood. If replicated, our findings also indicate that mothers in community samples are not at great risk for psychological distress that would compromise their capacity to utilize their EF while caring for their child. Future studies are called for, with a particular need for studies that examine these processes in fathers, as well as in samples of parents who face higher levels of exposure to chronic stressors and psychiatric symptoms.

## Data Availability Statement

The datasets presented in this article are not readily available because the medical faculty at the University of Turku has strict legal data sharing rules. The anonymized dataset is available upon request. Requests to access the datasets should be directed to statistician Juho Pelto, juho.pelto@utu.fi.

## Ethics Statement

The studies involving human participants were reviewed and approved by The Joint Ethics Committee of Turku University Hospital and University of Turku. Written informed consent to participate in this study was provided by the participants, or by the participants’ legal guardian.

## Author Contributions

EN: data collection, methodology, and writing – original draft. KD-D, MK, and ML: supervision, methodology, and writing – review and editing. E-LK: data collection, methodology, and writing – review and editing. EH and HH: data coding. EE: data collection. HK and LK: funding acquisition, methodology, and writing – review and editing. RK: supervision, funding acquisition, methodology, and writing – review and editing. All authors contributed to the article and approved the submitted version.

## Funding

This work was supported by the Academy of Finland [grant numbers: 253270, 134950, 286829, 308252 (RK), and 325292 Profi 5 (LK)], the Jane and Aatos Erkko Foundation, the Signe and Ane Gyllenberg Foundation (LK and RK), the State Research Funding of the Turku University Hospital (LK and RK), The Finnish Cultural Foundation (E-LK and RK), the Victoria Foundation (EN), and the Agneta and Carl-Erik Olin Foundation (EN). The funders had no role in study design, data collection and analysis, decision to publish, or preparation of the manuscript.

## Conflict of Interest

The authors declare that the research was conducted in the absence of any commercial or financial relationships that could be construed as a potential conflict of interest.

## Publisher’s Note

All claims expressed in this article are solely those of the authors and do not necessarily represent those of their affiliated organizations, or those of the publisher, the editors and the reviewers. Any product that may be evaluated in this article, or claim that may be made by its manufacturer, is not guaranteed or endorsed by the publisher.

## References

[ref1] Alink L. R. A. Mesman J. Van Zeijl J. Stolk M. N. Juffer F. Koot H. M.. (2006). The early childhood aggression curve: development of physical aggression in 10- to 50-month-old children. Child Dev. 77, 954–966. doi: 10.1111/j.1467-8624.2006.00912.x, PMID: 16942499

[ref2] Azar S. T. Reitz E. B. Goslin M. C. (2008). Mothering: thinking is part of the job description: application of cognitive views to understanding maladaptive parenting and doing intervention and prevention work. J. Appl. Dev. Psychol. 29, 295–304. doi: 10.1016/j.appdev.2008.04.009

[ref3] Ballesio A. Aquino M. R. J. V. Kyle S. D. Ferlazzo F. Lombardo C. (2019). Executive functions in insomnia disorder: a systematic review and exploratory meta-analysis. Front. Psychol. 10:101. doi: 10.3389/fpsyg.2019.00101, PMID: 30761049 PMC6363670

[ref4] Barrett J. Fleming A. S. (2011). Annual research review: all mothers are not created equal: neural and psychobiological perspectives on mothering and the importance of individual differences. J. Child Psychol. Psychiatry Allied Discip. 52, 368–397. doi: 10.1111/j.1469-7610.2010.02306.x 20925656

[ref45] Biringen Z. (2008). The Emotional Availability (EA) scales. 4th *Edn*. Boulder.

[ref5] Biringen Z. Derscheid D. Vliegen N. Closson L. Easterbrooks M. A. (2014). Emotional availability (EA): theoretical background, empirical research using the EA scales, and clinical applications. Dev. Rev. 34, 114–167. doi: 10.1016/j.dr.2014.01.002

[ref6] Biringen Z. Easterbrooks M. (2012). Emotional availability: concept, research, and window on developmental psychopathology. Dev. Psychopathol. 24, 1–8. doi: 10.1017/S0954579411000617, PMID: 22292989

[ref7] Bridgett D. J. Burt N. M. Edwards E. S. Deater-Deckard K. (2015). Intergenerational transmission of self-regulation: a multidisciplinary review and integrative conceptual framework. Psychol. Bull. 141, 602–654. doi: 10.1037/a0038662, PMID: 25938878 PMC4422221

[ref8] Chary M. McQuillan M. E. Bates J. E. Deater-Deckard K. (2020). Maternal executive function and sleep interact in the prediction of negative parenting. Behav. Sleep Med. 18, 203–216. doi: 10.1080/15402002.2018.1549042, PMID: 30585094 PMC6592784

[ref9] Cox J. L. Holden J. M. Sagovsky R. (1987). Detection of postnatal depression: development of the 10-item Edinburgh postnatal depression scale. Br. J. Psychiatry 150, 782–786. doi: 10.1192/bjp.150.6.782 3651732

[ref10] Crandall A. A. Deater-Deckard K. Riley A. W. (2015). Maternal emotion and cognitive control capacities and parenting: a conceptual framework. Dev. Rev. 36, 105–126. doi: 10.1016/j.dr.2015.01.004, PMID: 26028796 PMC4445866

[ref11] Deary I. J. Johnson W. (2010). Intelligence and education: causal perceptions drive analytic processes and therefore conclusions. Int. J. Epidemiol. 39, 1362–1369. doi: 10.1093/ije/dyq072, PMID: 20504860

[ref12] Deater-Deckard K. Wang Z. Chen N. Bell M. A. (2012). Maternal executive function, harsh parenting, and child conduct problems. J. Child Psychol. Psychiatry Allied Discip. 53, 1084–1091. doi: 10.1111/j.1469-7610.2012.02582.x, PMID: 22764829 PMC3460037

[ref13] Derogatis L. R. Lipman R. S. Covi L. (1973). SCL-90: an outpatient psychiatric rating scale--preliminary report. Psychopharmacol. Bull. 9, 13–28. PMID: 4682398

[ref14] Evans G. W. Li D. Whipple S. S. (2013). Cumulative risk and child development. Psychol. Bull. 139, 1342–1396. doi: 10.1037/a0031808, PMID: 23566018

[ref15] Friedman N. P. Miyake A. (2017). Unity and diversity of executive functions: individual differences as a window on cognitive structure. Cortex 86, 186–204. doi: 10.1016/j.cortex.2016.04.023, PMID: 27251123 PMC5104682

[ref16] Frigerio A. Nazzari S. (2021). Antenatal maternal anxiety, maternal sensitivity and toddlers’ behavioral problems: an investigation of possible pathways. Early Hum. Dev. 157:105364. doi: 10.1016/j.earlhumdev.2021.105364, PMID: 33813323

[ref17] Goodman J. H. (2004). Postpartum depression beyond the early postpartum period. J. Obstet. Gynecol. Neonatal. Nurs. 33, 410–420. doi: 10.1177/0884217504266915, PMID: 15346666

[ref18] Goodman J. H. Watson G. R. Stubbs B. (2016). Anxiety disorders in postpartum women: a systematic review and meta-analysis. J. Affect. Disord. 203, 292–331. doi: 10.1016/j.jad.2016.05.033, PMID: 27317922

[ref19] Hahn-Holbrook J. Cornwell-Hinrichs T. Anaya I. (2018). Economic and health predictors of national postpartum depression prevalence: a systematic review, meta-analysis, and meta-regression of 291 studies from 56 countries. Front. Psych. 8:248. doi: 10.3389/fpsyt.2017.00248 PMC5799244 29449816

[ref20] Hakanen H. Flykt M. Sinervä E. Nolvi S. Kataja E. L. Pelto J.. (2019). How maternal pre- and postnatal symptoms of depression and anxiety affect early mother-infant interaction? J. Affect. Disord. 257, 83–90. doi: 10.1016/j.jad.2019.06.048, PMID: 31299408

[ref21] Harris M. MacMillan H. Andrews K. Atkinson L. Kimber M. England-Mason G.. (2021). Maternal adverse childhood experiences, executive function & emotional availability in mother-child dyads. Child Abuse Negl. 111:104830. doi: 10.1016/j.chiabu.2020.104830, PMID: 33307519

[ref22] Karlsson L. Tolvanen M. Scheinin N. M. Uusitupa H. M. Korja R. Ekholm E.. (2018). Cohort profile: the FinnBrain birth cohort study (FinnBrain). Int. J. Epidemiol. 47, 15–16j. doi: 10.1093/ije/dyx173 29025073

[ref23] Korja K. McMahon C. (2021). Maternal prenatal mood problems and lower maternal emotional availability associated with lower quality of child’s emotional availability and higher negative affect during still-face procedure. Infancy doi: 10.1111/infa.12428 [Epub ahead of print] 34370394

[ref24] Krause A. J. Simon E. B. Mander B. A. Greer S. M. Saletin J. M. Goldstein-Piekarski A. N.. (2017). The sleep-deprived human brain. Nat. Rev. Neurosci. 18, 404–418. doi: 10.1038/nrn.2017.55, PMID: 28515433 PMC6143346

[ref25] MacMillan K. K. Lewis A. J. Watson S. J. Bourke D. Galbally M. (2021). Maternal social support, depression and emotional availability in early mother-infant interaction: findings from a pregnancy cohort. J. Affect. Disord. 292, 757–765. doi: 10.1016/j.jad.2021.05.048, PMID: 34167025

[ref26] MacMillan K. K. Lewis A. J. Watson S. J. Galbally M. (2020). Maternal depression and the emotional availability of mothers at six months postpartum: findings from the mercy pregnancy and emotional wellbeing study (MPEWS) pregnancy cohort. J. Affect. Disord. 266, 678–685. doi: 10.1016/j.jad.2020.01.109, PMID: 32056944

[ref27] Madigan S. Prime H. Graham S. A. Rodrigues M. Anderson N. Khoury J.. (2019). Parenting behavior and child language: a meta-analysis. Pediatrics 144:e20183556. doi: 10.1542/peds.2018-3556 31551396

[ref28] Mindell J. A. Sadeh A. Kwon R. Goh D. Y. T. (2015). Relationship between child and maternal sleep: a developmental and cross-cultural comparison. J. Pediatr. Psychol. 4, 689–696. doi: 10.1093/jpepsy/jsv008 25749896

[ref29] Miyake A. Friedman N. P. Emerson M. J. Witzki A. H. Howerter A. Wager T. D. (2000). The unity and diversity of executive functions and their contributions to complex “frontal lobe” tasks: a latent variable analysis. Cogn. Psychol. 41, 49–100. doi: 10.1006/cogp.1999.0734, PMID: 10945922

[ref30] Nordenswan E. Kataja E. L. Deater-Deckard K. Korja R. Karrasch M. Laine M.. (2020). Latent structure of executive functioning/learning tasks in the cogstate computerized battery. SAGE Open 10:2158244020948846. doi: 10.1177/2158244020948846

[ref31] Payne V. G. Isaacs L. D. (2017). Human Motor Development: A Lifespan Approach. New York: Routledge.

[ref32] Pietrzak R. H. Olver J. Norman T. Piskulic D. Maruff P. Snyder P. J. (2009). A comparison of the cogstate schizophrenia battery and the measurement and treatment research to improve cognition in schizophrenia (MATRICS) battery in assessing cognitive impairment in chronic schizophrenia. J. Clin. Exp. Neuropsychol. 31, 848–859. doi: 10.1080/13803390802592458, PMID: 19142774

[ref33] Porreca A. Biringen Z. Parolin M. Saunders H. Ballarotto G. Simonelli A. (2018). Emotional availability, neuropsychological functioning, and psychopathology: the context of parental substance use disorder. Biomed. Res. Int. 2018:5359037. doi: 10.1155/2018/5359037, PMID: 29888268 PMC5985126

[ref34] Rossen L. Mattick R. P. Wilson J. Clare P. J. Burns L. Allsop S.. (2019). Mother–infant bonding and emotional availability at 12-months of age: the role of early postnatal bonding, maternal substance use and mental health. Matern. Child Health J. 23, 1686–1698. doi: 10.1007/s10995-019-02809-1, PMID: 31529248

[ref35] Salo S. Flykt M. Mäkelä J. Lassenius-Panula L. Korja R. Lindaman S.. (2020). The impact of theraplay^®^ therapy on parent-child interaction and child psychiatric symptoms: a pilot study. Int. J. Play 9, 331–352. doi: 10.1080/21594937.2020.1806500

[ref36] Saunders H. Kraus A. Barone L. Biringen Z. (2015). Emotional availability: theory, research, and intervention. Front. Psychol. 6:1069. doi: 10.3389/fpsyg.2015.01069, PMID: 26283996 PMC4516809

[ref37] Schmitz N. Hartkamp N. Franke G. H. (2000). Assessing clinically significant change: application to the SCL-90-R. Psychol. Rep. 86, 263–274. doi: 10.2466/pr0.2000.86.1.263, PMID: 10778279

[ref38] Smith-Nielsen J. Matthey S. Lange T. Væver M. S. (2018). Validation of the Edinburgh postnatal depression scale against both DSM-5 and ICD-10 diagnostic criteria for depression. BMC Psychiatry 18, 1–12. doi: 10.1186/s12888-018-1965-7, PMID: 30572867 PMC6302501

[ref39] Snyder H. R. Miyake A. Hankin B. L. (2015). Advancing understanding of executive function impairments and psychopathology: bridging the gap between clinical and cognitive approaches. Front. Psychol. 6:328. doi: 10.3389/fpsyg.2015.00328, PMID: 25859234 PMC4374537

[ref40] Soldatos C. R. Dikeos D. G. Paparrigopoulos T. J. (2000). Athens insomnia scale: validation of an instrument based on ICD-10 criteria. J. Psychosom. Res. 48, 555–560. doi: 10.1016/S0022-3999(00)00095-7, PMID: 11033374

[ref41] Soldatos C. R. Dikeos D. G. Paparrigopoulos T. J. (2003). The diagnostic validity of the Athens insomnia scale. J. Psychosom. Res. 55, 263–267. doi: 10.1016/S0022-3999(02)00604-9, PMID: 12932801

[ref42] Sturge-Apple M. L. Suor J. H. Skibo M. A. (2014). Maternal child-centered attributions and harsh discipline: the moderating role of maternal working memory across socioeconomic contexts. J. Fam. Psychol. 28, 645–654. doi: 10.1037/fam0000023, PMID: 25221969 PMC4318501

[ref43] Teti D. M. Shimizu M. Crosby B. Kim B.-R. (2016). Sleep arrangements, parent-infant sleep during the first year, and family functioning. Dev. Psychol. 52, 1169–1181. doi: 10.1037/dev0000148, PMID: 27389833 PMC4959950

[ref44] Wachs T. D. Georgieff M. Cusick S. Mcewen B. S. (2014). Issues in the timing of integrated early interventions: contributions from nutrition, neuroscience, and psychological research. Ann. N. Y. Acad. Sci. 1308, 89–106. doi: 10.1111/nyas.12314, PMID: 24354763 PMC4075015

